# 

**Published:** 1843-12-30

**Authors:** 


### CONTENTS.

*Clinical Lectures and Reports.—University of Pennsylvania.*

On Consecutive Syphilis. By Dr. Clymer. Lecture II, - - - - - - 301

A List of Cases Discharged from the Surgical Wards of the Pennsylvania Hospital, during the month of November, 1843. Reported by Ellerslie Wallace, M. D., Resident Surgeon, - - - - - - 304

*Bibliographical Notices.*

Review of Dr. Caldwell's pamphlet, entitled Physiology Vindicated, in a Critique on Liebig's Chemistry. By Robert Peter, M.D., Professor of Chemistry and Pharmacy, in Transylvania University, - - - - 306

On the Nature and Treatment of Renal Diseases; being an inquiry into the connection of Diabetes, Calculus, and other affections of the Kidney and Bladder, with Indigestion. By William Prout, M. D., F. R. S., etc. From the Fourth Revised London Edition, with Plates, - - - - 206

*Retrospect of the Medical Sciences.*

On the successful employment of Belladonna as a prophylactic during the prevalence of the Epidemic Scarlatina. By M. Stievenart, of Valenciennes, - - - - 307

A Clinical Lecture on Aneurism of the thigh. By Robert Liston, Surgeon, F. R. S. - 308

Doctor Gregory on Small-pox, - - 310

Advantage of Medicines in a Liquid Form, 312

Seat of Tubercles, - - - - 312

French Accoucheurs, - - - - 312

Abscess of the Lung, - - - - 312

